# “Alternating” the Diagnosis after 40 Years of Disease: The Thousand Faces of ATP1A3 Mutation

**DOI:** 10.1002/mdc3.13980

**Published:** 2024-01-22

**Authors:** Silvia Gallo, Fabienne Ory‐Magne, Clémence Leung, Margherita Fabbri, Bastien Estublier, Emmanuel Cheuret, Olivier Patat, Raquel Pinheiro Barbosa

**Affiliations:** ^1^ Department of Neurology University Hospital of Toulouse Toulouse France; ^2^ Neurology Unit, Department of Translational Medicine University of Piemonte Orientale Novara Italy; ^3^ French Reference Center for MSA, Centre d'Investigation Clinique de Toulouse CIC1436, Department of Neurosciences and Clinical Pharmacology, NS‐Park/FCRIN Network, NeuroToul COEN Center University Hospital of Toulouse, INSERM, University of Toulouse 3 Toulouse France; ^4^ Neuropediatric Department Toulouse‐Purpan University Hospital Toulouse France; ^5^ Department of Medical Genetics Toulouse University Hospital Toulouse France

A wide range of clinical phenotypes has been associated with ATP1A3 gene mutations, showing phenotypic variability and occasional overlap.^1^ High phenotypic variability, lack of specific symptoms and acute presentation with rapid symptom progression can lead to a diagnostic delay.^2^

## Case Report

A 42‐year‐old woman presented to our movement disorder department with ataxic gait with dystonic features evolving for several years. Birth and neurodevelopmental history were normal until the age of 24 months when, following a sore throat, she developed an acute encephalitis‐like event characterized by fever, epileptic seizures, reduced vigilance, diffuse hypotonia with absent reflexes and swallowing difficulties. Despite a normal MRI and lumbar puncture, she was diagnosed with autoimmune encephalitis and treated with steroids and immunosuppressive therapy without improvement. Although vigilance improved spontaneously, she developed moderate gait impairment with ataxic features and involuntary movements. Her clinical picture remained stable over the years, with symptoms being interpreted as a sequela of autoimmune encephalitis. Neurological examination at age 42 revealed cerebellar features, including an ataxic broad‐based gait with dystonic features, upper and lower limbs dysmetria, scanning speech and motor impersistence at tongue protrusion. Distal choreodystonic movements in all four limbs with facial dyskinesias were also observed. Neuroophthalmological evaluation revealed slow and hypermetric saccades without gaze palsy (Video 1). Neuropsychological evaluation, MRI (Fig. S1) hearing test and fundoscopic findings were all normal.

**Video 1:** Neurological examination of patient #1.

At the age of 40, her 12‐month‐old son, developed an encephalitis‐like event following an otitis media. He was born after an uneventful pregnancy and no neurodevelopment delay was noticed until then. He presented with fever, decreased vigilance, diffuse hypotonia with absent reflexes, difficulty swallowing, and a left‐side hemiplegia. Blood tests, neurophysiological tests and neuroimaging showed no abnormalities. Bickerstaff encephalitis was diagnosed and a treatment with intravenous immunoglobulins was started without benefit. Hemiplegia, vigilance and tone improved spontaneously, but an ataxic gait, head titubation and scanning speech persisted (Video 2).

**Video 2:** Neurological findings of patient #2.

This event prompted a reevaluation of the mother's diagnosis.

Both cases initially led to the diagnosis of autoimmune encephalitis. However, the similarity in the two cases raised suspicion of a potential genetic disease, particularly a channelopathy like ATP1A3 mutation.

Genetic testing revealed a heterozygous p.Arg756Cys mutation in the ATP1A3 gene. This finding led to the diagnosis of remitting encephalopathy with cerebellar ataxia and fever‐induced paroxysmal weakness and encephalopathy (RECA/FIPWE) in the mother. In the son it resulted in the same encephalopathy overlapping with alternating childhood hemiplegia.

The mother never presented a relapse, but at the age of 4, the son developed a right‐side hemiplegia with swallowing difficulties following an HHV6 infection, leaving severe sequelae: loss of independent gait due to a severe cerebellar syndrome and significant dysarthria. To minimize the risk of further relapses, flunarizine was started.^3^, ^4^

## Discussion

Mutations in the α3 catalytic subunit of Na+/K+ adenosine triphosphatases, encoded by ATP1A3, are classically associated with three distinct neurological syndromes: rapid‐onset dystonia parkinsonism (RDP); alternating hemiplegia of childhood (AHC); and cerebellar ataxia, areflexia, pes cavus, optic atrophy, and sensorineural hearing loss (CAPOS). However, recent research expanded the list of associated phenotypes, some lacking alternating hemiplegia, making early diagnosis challenging.^2^

In our first patient, no hemiplegic event was observed during the initial acute episode, initially ruling out an ATP1A3 mutation diagnosis according to the classic AHC criteria.^5^ The diagnosis was only revisited when her son presented with an acute‐like event with hemiplegia. Her clinical picture had other atypical features that precluded an earlier diagnosis, such as choreic movements and the absence of the classic waxing and waning pattern, with multiple episodes of paroxysmal events.

The rising prevalence of atypical phenotypes underscores the necessity for more comprehensive diagnostic criteria to prevent delays in identifying ATP1A3 mutations in patients with atypical or incomplete presentations. In line with this, a broader set of symptoms has been proposed for diagnosing an ATP1A3‐related disorder.^6^ Our first patient would meet these comprehensive criteria, even though her presentation differed from classical ATP1A3 mutations.

In addition to the increasing number of phenotypes, the presence of multiple phenotypes in one patients, with AHC features evolving into other ATP1A3‐related syndromes during follow‐up, argues against the notion of distinct syndromes and supports a more flexible diagnostic approach.^2^, ^7^

Despite sharing the same mutation, the clinical phenotypes of both patients did not completely overlap. The p.Arg756Cys mutation is a likely pathogenic mutation of the ATP1A3 gene^8^ and it is typically associated with the RECA/FIPWE phenotype. The diagnosis of the first patient, while the patient's son also presented an AHC‐phenotype, is not commonly associated with this mutation. Once more, this emphasizes a diagnosis based on a spectrum of symptoms over a rigid phenotyping approach.^2^

A combination of broader symptom categories, such as paroxysmal symptoms, hyperkinetic symptoms, neuropsychiatric symptoms, and cognitive impairment, has been proposed to increase sensitivity in identifying patients with ATP1A3‐related disorders than relying on restricted phenotypes.^1^

This case highlights the phenotypic heterogeneity of ATP1A3 mutations, emphasizing the need to consider a combination of symptoms across different categories instead of strictly classifying ATP1A3 disorders as separate syndromes.

## DISCLOSURES

**Ethical Compliance Statement:** Written consent was obtained from the patients before manuscript writing. The authors confirm that the approval of an institutional review board was not required for this work. We confirm that we have read the Journal's position on issues involved in ethical publication and affirm that this work is consistent with those guidelines.

**Funding Sources and Conflicts of Interest:** The authors declare no conflict of interest concerning the research related to the manuscript, no relevant conflicts of interests/financial disclosure.

**Financial Disclosures for the previous 12 months:** SG, CL, BE, EC and OP report no additional disclosures. FO‐M has received honoraria for serving as an advisory board member from Abbvie, Medtronic, Orphalan and Orkyn, and for consultancy activities from Aguettant, Abbvie, Orphalan, Homeperf and Orkyn. MF reports honoraria to speak from BIAL and AbbVie.

## Author Roles

(1) Conception of the work: A. Organization, B. Execution; (2) Manuscript: A. Writing of the first draft, B. Review and Critique.

S.G.: 1A, 1B, 2A.

F.O‐M., C.L., M.F.: 1A, 2B.

B.E., E.C., O.P.: 2B.

R.P.B.: 1A, 1B, 2A, 2B.

## Supporting information


**Figure S1.** MRI findings of the #1 patient showing mild cortical atrophy without significant cerebellar atrophy.
