# Supplementary figures and images for: “Alternating” the Diagnosis after 40 Years of Disease: The Thousand Faces of ATP1A3 Mutation
Source: Mov Disord Clin Pract. 2024 Jan 22;11(Suppl 2):S11–3. doi: 10.1002/mdc3.13980 (PMC11322589; doi:10.1002/mdc3.13980)

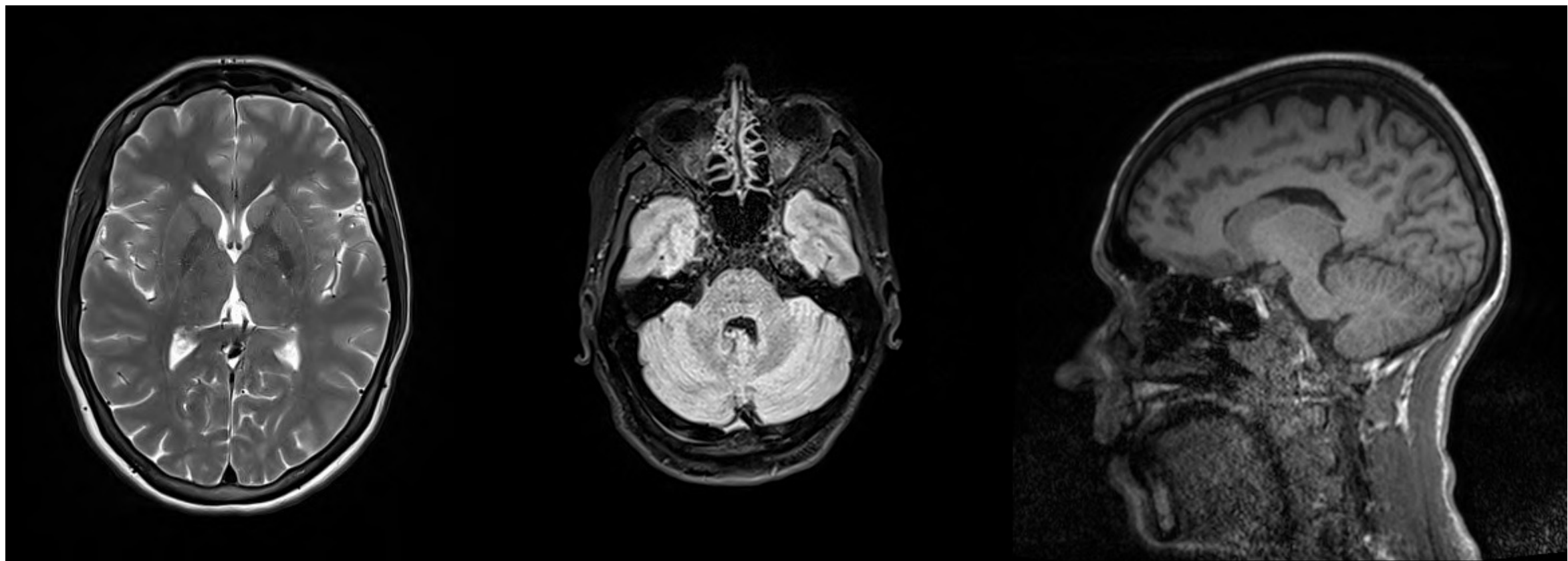

Supplement: Supplementary file 1 — Figure S1. MRI findings of the #1 patient showing mild cortical atrophy without significant cerebellar atrophy. [file MDC3-11-S11-s001.pdf]
